# High frequency of NDM-1 and OXA-48 carbapenemase genes among *Klebsiella pneumoniae* isolates in central Iran

**DOI:** 10.1186/s12866-023-02840-x

**Published:** 2023-04-11

**Authors:** Elnaz Abbasi, Ehsanollah Ghaznavi-Rad

**Affiliations:** 1 Department of Microbiology & Immunology, Khomein University of Medical Sciences, Khomein, Iran; 2 Molecular and Medicine Research Center, Faculty of Medicine, Arak University of Medical Sciences, Arak, Iran; 3 Department of Microbiology & Immunology, Faculty of Medicine, Arak University of Medical Sciences, Arak, Iran

**Keywords:** Multidrug-resistant, Carbapenem-resistant *K. pneumoniae*, *bla*_OXA−48_, *bla*_NDM_, Integron, Iran

## Abstract

**Background:**

The emergence and distribution of multidrug-resistant (MDR) and carbapenem-resistant *Klebsiella pneumoniae* (CRKP) has become a global health threat. Therefore, this study aimed to investigate the frequency and antibiotic resistance patterns of MDR, extensively drug-resistant (XDR), and CRKP, as well as the antibiotic resistance genes of *Klebsiella pneumoniae* (*K. pneumoniae*) isolates from patients’ infectious samples from central Iran.

**Methods:**

This study examined 546 clinical samples of patients to identify *K. pneumoniae.* The isolates were investigated for their antibiotic resistance profile, extended-spectrum β-lactamase (ESBL), AMPC β-lactamase, carbapenemase resistance, sulfonamide, tetracycline, plasmid-mediated quinolone resistance (PMQR) along with their resistance genes, integrase, and quaternary ammonium compounds (*qac*) by polymerase chain reaction (PCR).

**Results:**

Out of 546 clinical samples, 121 (22.1%) cases of *K. pneumoniae* were identified using culture and PCR methods. The highest antibiotic resistance rates were found for ampicillin (119/121; 98.3%), cotrimoxazole (78/121; 64.4%), and cefixime, cefotaxime, ceftriaxone, and ceftazidime as a group (77/121; 63.6%). Tigecycline, colistin, and fosfomycin were the most effective antimicrobial agents with 98.4%, 96.7%, and 95.9% susceptibility, respectively. The amount of CRKP was 51 (42.1%). All CRKP isolates were MDR. The most abundant genes were *bla*_TEM_ (77/77; 100%), *bla*_CTX−M1_ (76/77; 98.7%), *bla*_SHV_ (76/77; 98.7%), *bla*_CTX−M15_ (73/77; 94.8%) for ESBL; *bla*_CIT_ 28 (48.3%) and *bla*_CMY−2_ 26 (44.8%) for AMPC β-lactamase; and *bla*_OXA−48_ 46 (90.1%) and *bla*_NDM_ 36 (70.5%) for carbapenemase. Among the PMQR determinants, *qnrB* (25/52; 48%), *qnrS* (19/52; 36.5%), and *qnrA* (11/52; 21.1%) were positive from the isolates. *TetA* and *tetB* were recognized in 25 (44.6%) and 17 (30.3%) isolates, respectively. Class 1 and 2 integrons were recognized in 97 (80.1%) and 53 (43.8%) isolates, respectively.

**Conclusions:**

Due to the high prevalence of MDR and CRKP in central Iran, tracking and immediate intervention are necessary for control and inhibition of *K. pneumoniae* resistant isolates. Tigecycline, colistin, and fosfomycin are the best treatment options for treatment of patients with CRKP in this geographical area.

## Background

*Klebsiella pneumoniae* (*K. pneumoniae*) is one of the most important causes of urinary, respiratory, sepsis, and wound infections in communities and hospitals [1, 2]. Multidrug-resistant (MDR) and extensively drug-resistant (XDR) *K. pneumoniae* are a leading cause of healthcare-associated infections, which are linked to rising medical expenditures and increased morbidity and mortality [3, 4]. The emergence and dissemination of MDR and XDR *K. pneumoniae* isolates pose a significant threat to infection control programs and, therefore, require immediate attention [4, 5]. Carbapenems are the last-line for treatment to treat serious infections with MDR *K. pneumonia* [5]. Unfortunately, Carbapenem-resistant *K. pneumoniae* (CRKP) is on the rise worldwide and is of especial clinical concern globally as such infections are a challenge to treat [6]. In particular, CRKP is considered a difficult-to-treat organism as there are few therapeutic options [3]. Therefore, it also results in high mortality [6]. The most clinically significant carbapenemase genes in CRKP are Ambler class A β-lactamases (*bla*_KPC_), class B metallo-β-lactamases (MBLs) (*bla*_NDM_ and *bla*_VIM_), and class D β-lactamases (*bla*_OXA−48_) [6]. The release of *bla*_OXA−48_ and *bla*_NDM−1_ producing *K. pneumoniae* causes great concern because they have a significant ability to spread [7]. Combination antibiotics often used in the treatment of CRKP include the following: colistin, in combination with tigecycline or rifampin or carbapenem; fosfomycin plus colistin or amikacin; and double-carbapenem antibiotics (a combination of doripenem and ertapenem) [5].

Since the discovery of *bla*_OXA−48_ and *bla*_NDM_ carbapenemases in Turkey and India in 2001 and 2008, respectively, these strains have been implicated in a large number of nosocomial outbreaks in other parts of the world, including the Middle East (e.g., Iran) [3–5].

Given the diffusion of different resistances (extended-spectrum β-lactamase (ESBL), MDR, and carbapenem) in *K. pneumoniae* on both national and international levels and the lack of data on their frequency in this geographical area, we aimed at investigating the frequency of *K. pneumoniae* and the phenotypic and genotypic level of MDR, XDR, and carbapenem-resistant *K. pneumoniae* in patients with infections from central Iran.

## Materials and methods

### Sample collection

A statement to confirm that all methods were carried out in accordance with relevant guidelines and regulations. Must include a sentence confirming that informed consent was obtained from all subjects and/or their legal guardian(s). This study protocol was approved by the Ethics Committee of the Arak University of Medical Sciences (ARAKMU.REC.1396.3.7). For this cross-sectional, descriptive study, 546 clinical samples of urine, tracheal aspirates, wound, and blood were collected from adult inpatients in the Imam Khomeini Hospital (Khomein, Iran) from April 2017 to March 2019. Patients had not received antibiotics before the sampling.

#### Phenotypic investigation

*K. pneumoniae* isolates were identified by Gram stain and conventional biochemical tests (Triple Sugar Iron(TSI), Simmons Citrate agar, SIM, Urea agar, Lysine Iron Agar (LIA), Methyl Red / Voges-Proskauer (MR/VP), Oxidative fermentative (OF)), and they were confirmed by application programming interface (API) testing (bioMérieux, France) [8]. *K. pneumoniae* ATCC 700,603 and *Escherichia coli* ATCC 25,922 were used as controls in each assay (acquired from the Microbiology Department of the Arak University of Medical Sciences).

#### Investigating *K. pneumoniae* antibiotic resistance by disk diffusion

Using the Clinical and Laboratory Standards Institute (CLSI) guidelines [9], an antibiogram assay was performed on the isolated *K. pneumoniae* colonies. The antibiotic discs contained ampicillin (30 µg), cotrimoxazole (25 µg), cefixime (5 µg), cefotaxime (30 µg), ceftriaxone (30 µg), ceftazidime (30 µg), gentamicin (10 µg), amikacin (30 µg), tetracycline (30 µg), doxycycline (30 µg), minocycline (30 µg), tigecycline (15 µg), ciprofloxacin (5 µg), levofloxacin (5 µg), ampicillin/sulbactam (100/10 µg), piperacillin/tazobactam (100/10 µg), imipenem (10 µg), meropenem (10 µg), ertapenem (10 µg), doripenem (10 µg), aztreonam (30 µg), colistin (10 µg), and fosfomycin (200 µg) (Mast Diagnostics, United Kingdom) [4, 10].

The minimum inhibitory concentration (MIC) of imipenem, meropenem, and colistin for isolates resistant to carbapenems was determined by using E-test (Liofilchem, Italy) according to the 2021 CLSI guidelines [9].

#### Detection of ESBL and AmpC β-lactamases by phenotypic methods

To recognize ESBL-positive isolates, the specimens were subjected to combination disk diffusion [11] and double-disk synergy testing procedures [12], and to recognize AmpC-positive isolates, disk testing and phenol boronic acid procedures were used [13].

#### Detection of carbapenemase

To recognize carbapenemase-positive isolates, the specimens were subjected to the modified Hodge test (MHT), modified carbapenem inactivation method (mCIM), and EDTA-modified carbapenem inactivation method (eCIM) procedures according to the 2021 CLSI guidelines [9, 14].

## Genotypic investigations

### DNA extraction

DNA was extracted from the *K. pneumoniae* isolates using a QIAamp DNA mini kit (Qiagen, Hilden, Germany) in accordance with the manufacturer’s protocol.

All culture-positive samples were confirmed to be positive using the *16s RNA* primers in the PCR method (Table 1) [15]. The ESBL (*bla*_*TEM*_, *bla*_*SHV*_, and *bla*_*CTX−M−1*_, _*2*_, _*8*_, _*15*_), AmpC (*bla*_*CIT*_, *bla*_*CMY−2*_, *bla*_*ACC*_, *bla*_*FOX*_, *bla*_*MOX*_, and *bla*_*DHA*_), and carbapenemase genes [(Ambler class A: *bla*_*KPC*_ and *bla*_*GES*_), (Ambler class B: MBLs: *bla*_*NDM*_, *bla*_*VIM*_, *bla*_*SIM*_, *bla*_*GIM*_, *bla*_*SPM*_, and *bla*_*IMP*_) (Ambler class D: *bla*_*Oxa−48*_)] were recognized by PCR [16, 17]. Genes *sul1* and *2* for sulfonamide resistance and *tet(A)* and *(B)* genes for tetracycline resistance were recognized by PCR as well [18]. To amplify the PMQR targets, PCR of the *qnr* determinant genes *qnrA*, *qnrB*, and *qnrS* was performed [19]. Mutations in the *parC* and *gyrA* genes of the quinolone-resistant *K. pneumoniae* isolates were determined using DNA sequencing [18]. Quaternary ammonium compound (QAC) resistance genes were identified using PCR (Table 1) [18].

**Table 1:** The primers used in this study

Target gene description		Primer	Sequence 5’→3’	Amplicon size (bp)	Annealing Temperature	References
*K.pneumoniae*		*16s-rRNA*-F *16s-rRNA*-R	5-ATTTGAAGAGGTTGCAAACGAT-3 5-TTCACTCTGAAGTTTTCTTGTGTTC-3	130	54	[15]
β-Lactamase ESBL+		*TEM*-F *TEM*-R	5-AAAGATGCTGAAGATCA-3 5-TTTGGTATGGCTTCATTC-3	425	44	[18]
		*SHV*-F *SHV*-R	5-GCGAAAGCCAGCTGTCGGGC-3 5-GATTGGCGGCGCTGTTATCGC-3	304	62	[18]
		*CTX-M1*-F *CTX-M1*-R	5-AAGACTGGGTGTGGCATTGA-3 5-AGGCTGGGTGAAGTAAGTGA-3	670	52	[18]
		*CTX-M2*-F *CTX-M2*-R	5-CGACGCTACCCCTGCTATT-3 5-CCAGCGTCAGATTTTTCAGG-3	552	60	[18]
		*CTX-M8*-F *CTX-M8*-R	5-CGCTTTGCCATGTGCAGCACC-3 5-GCTCAGTACGATCGAGCC-3	307	55	[18]
		*CTX-M15*-F *CTX-M15*-R	5-CACACGTGGAATTTAGGGACT-3 5-GCCGTCTAAGGCGATAAACA-3	955	55	[18]
β-Lactamase AmpC+		*CMY-2*-F *CMY-2*-R	5′-GCACTTAGCCACCTATACGGCAG-3′ 5′-GCTTTTCAAGAATGCGCCAGG-3′	758	58	[18]
		*Fox*-F *Fox*-R	5-AACATGGGGTATCAGGGAGATG-3 5-CAAAGCGCGTAACCGGATTGG-3	190	64	[18]
		*Mox*-F *Mox*-R	5-GCTGCTCAAGGAGCACAGGAT-3 5-CACATTGACATAGGTGTGGTGC-3	520	64	[18]
		*DHA*-F *DHA*-R	5-AACTTTCACAGGTGTGCTGGGT-3 5-CCGTACGCATACTGGCTTTGC-3	405	64	[18]
		*ACC*-F *ACC*-R	5-AACAGCCTCAGCAGCCGGTTA-3 5-TTCGCCGCAATCATCCCTAGC-3	346	64	[18]
		*CIT*-F *CIT*-R	5-TGGCCAGAACTGACAGGCAAA-3 5-TTTCTCCTGAACGTGGCTGGC-3	462	64	[18]
Carbapenemase	Ambler class A blactamases	*KPC*-F *KPC*-R	5-CGTCTAGTTCTGCTGTCTTG-3 5-CTTGTCATCCTTGTTAGGCG-3	798	52	[16]
		*GES*-F *GES*-R	5-ATGCGCTTCATTCACGCAC-3 5-CTATTTGTCCGTGCTCAGG-3	846	52	[17]
	Ambler class B metallo- β -lactamases (MBLs)	*NDM*-F *NDM*-R	5-GGTTTGGCGATCTGGTTTTC-3 5-CGGAATGGCTCATCACGATC-3	621	52	[16]
		*VIM*-F *VIM*-R	5-GATGGTGTTTGGTCGCATA-3 5-CGAATGCGCAGCACCAG-3	390	52	[16]
		*SIM*-F *SIM*-R	5-TACAAGGGATTCGGCATCG-3 5-TAATGGCCTGTTCCCATGTG-3	570	52	[16]
		*GIM*-F *GIM*-R	5-TCGACACACCTTGGTCTGAA-3 5-AACTTCTGGGTACGCAAACG-3	477	52	[16]
		*SPM*-F *SPM*-R	5-AAAATCTGGGTACGCAAACG-3 5-ACATTATCCGCTGGAACAGG-3	271	52	[16]
		*IMP*-F *IMP*-R	5-GGAATAGAGTGGCTTAAYTCTC-3 5-GGTTTAAYAAAACAACCACC-3	232	52	[16]
	Ambler class D b-lactamases	*Oxa-48*-F *Oxa-48*-R	5-GCGTGGTTAAGGATGAACAC-3 5-CATCAAGTTCAACCCAACCG-3	438	52	[16]
*Fluoroquinolone*		*gyrA*-F *gyrA*-R	5-AAATCTGCCCGTGTCGTTGGT-3 5-GCCATACCTACGGCGATACC-3	344	55	[19]
		*parC*-F *parC*-R	5-CTGAATGCCAGCGCCAAATT-3 5-GCGAACGATTTCGGATCGTC-3	168	55	[19]
		*qnrS*-F *qnrS*-R	5-TGGAAACCTACAATCATACATATCG-3 5-TTAGTCAGGATAAACAACAATACCC-3	656	60	[19]
		*qnrA*-F *qnrA*-R	5-GATAAAGTTTTTCAGCAAGAGG-3 5-ATCCAGATCGGCAAAGGTTA-3	593	60	[19]
		*qnrB*-F *qnrB*-R	5-GTTGGCGAAAAAATTGACAGAA-3 5-ACTCCGAATTGGTCAGATCG-3	264	53	[19]
Tetracycline		*tetA*-F *tetA*-R	5-GTGAAACCCAACATACCCC-3 5-GAAGGCAAGCAGGATGTAG-3	888	55	[20]
		*tetB*-F *tetB*-R	5-CCTTATCATGCCAGTCTTGC-3 5-ACTGCCGTTTTTTCGCC-3	774	55	[20]
Integrase1		*Int1*-F *Int1*-R	5-CAGTGGACATAAGCCTGTTC-3 5-CCCGAGGCATAGACTGTA-3	160	55	[21]
Integrase2		*Int2*-F *Int2*-R	5-TTGCGAGTATCCATAACCTG-3 5-TTACCTGCACTGGATTAAGC-3	288	55	[21]
Integrase3		*Int3*-F *Int3*-R	5-GCCTCCGGCAGCGACTTTCAG-3 5-ACGGATCTGCCAAACCTGACT-3	979	59	[21]
Sulfonamide resistance		*Sul1*-F *Sul1*-R	5-TCACCGAGGACTCCTTCTTC-3 5-CAGTCCGCCTCAGCAATATC-3	331	65	[18]
		*Sul2*-F *Sul2*-R	5-CCTGTTTCGTCCGACACAGA-3 5-GAAGCGCAGCCGCAATTCAT-3	435	58	[18]
Quaternary ammonium compounds		*qac*-F *qac*-R	5-GCCCTACACAAATTGGGAGA-3 5-CTGCGGTACCACTGCCACAA-3	370	55	[22]

### Integron detection

To check class 1, 2, and 3 integrons, a PCR assay was performed as shown in Table 1.

## Results

### Phenotypic and genotypic investigation

Out of the 546 infectious samples, 121 (22.1%) were identified as *K. pneumoniae* using the culture method. All culture-positive samples were confirmed to be positive using the *16s RNA* primers in the PCR method (Table 1). The average age of the *K. pneumoniae* patients was 47 years and 3 months. Of the 121 patients included in the study, 82 (67.7%) males and 39 (32.2%) females were infected by *K. pneumoniae*, yielding an infection ratio of males to females of 2.1:1. The most infectious samples of *K. pneumoniae* were seen in urine (62, 51.2%), respiratory (48, 39.6%), wound (11, 9%), and blood infections (8, 6.6%).

### Phenotypic and genotypic antibiotic resistance determination

Using the CLSI 2021 guidelines, the highest resistance rates of *K. pneumoniae* were obtained against ampicillin (119/121, 98.3%), cotrimoxazole (78/121, 64.4%), cefixime (77/121, 63.6%), cefotaxime (77/121, 63.6%), ceftriaxone (77/121, 63.6%), ceftazidime (77/121, 63.6%), and gentamicin (74/121, 61.1%) (Table 2). Of the *K. pneumoniae* isolates, 77 were ESBL-positive. All ESBL-positive isolates contained *bla*_*TEM*_ resistance genes (Table 2). The most frequent ESBL genes were *bla*_TEM_ (77, 100%), *bla*_CTX−M1_ (76, 98.7%), *bla*_SHV_ (76, 98.7%), and *bla*_CTX−M15_ (73, 94.8%). The most frequent AmpC genes were *bla*_CIT_ (28, 48.3%) and *bla*_CMY−2_ (26, 44.8%) (Table 2).

**Table 2:** Phenotypic and Genotypic antibiotic resistance rates in *K. pneumoniae*

Antibiotic	*K. pneumoniae* n:121	Resistance		Target gene	Frequency of resistance genes	Resistance	Target gene	Frequency of resistance genes
Ampicillin	119(98.3%)							
Cotrimoxazole	78 (64.4%)	Sulfonamide		*sul1*	55(70.5%)			
				*sul2*	32(41%)			
Cefixime	77 (63.6%)	ESBL+		*bla*_TEM_	77(100%)	n:58* AmpC+	*bla*_CIT_	28(48.3%)
Cefotaxime	77 (63.6%)			*bla*_CTX−M1_	76(98.7%)		*bla*_CMY−2_	26(44.8%)
				*bla*_SHV_	76(98.7%)		*bla*_ACC_	4(6.9%)
Ceftriaxone	77 (63.6%)			*bla*_CTX−M15_	73(94.8%)		*bla*_FOX_	0%
Ceftazidime	77 (63.6%)			*bla*_CTX−M2_	0%		*bla*_MOX_	0%
				*bla*_CTX−M8_	0%		*bla*_DHA_	0%
Gentamicin	74(61.1%)							
Amikacin	41(33.8%)							
Tetracycline	56(46.3%)	Tetracycline		*tetA*	25(44.6%)			
Doxycycline	56(46.3%)							
Minocycline	56(46.3%)			*tetB*	17(30.3%)			
Tigecycline	2(1.6%)							
Ciprofloxacin	52(43%)	*Fluoroquinolone*		*gyrA*	27(51.9%)			
				*qnrB*	25(48%)			
Levofloxacin	52(43%)			*qnrS*	19(36.5%)			
				*qnrA*	11(21.1%)			
				*parC*	0%			
Ampicillin/sulbactam	58(47.9%)							
Piperacillin/tazobactam	55(45.4%)							
Imipenem	50(41.3%)	Carbapenemase	Ambler class A blactamases	*bla*_GES_	11(21.5%)			
				*bla*_KPC_	0%			
Meropenem	51(42.1%)		Ambler class B metallo- β -lactamases (MBLs)	*bla*_NDM_	36(70.5%)			
				*bla*_VIM_	9(17.6%)			
Doripenem	51(42.1%)			*bla*_SIM_	0%			
				*bla*_GIM_	0%			
				*bla*_IMP_	0%			
				*bla*_SPM_	0%			
			Ambler class D b-lactamases	*bla*_Oxa−48_	46(90.1%)			
Ertapenem	51(42.1%)							
Aztreonam	50(41.3%)							
Colistin	4(3.3%)							
Fosfomycin	5(4.1%)							
		Integrase		*int1*	97(80.1%)			
				*int2*	53(43.8%)			
				*int3*	0%			
		Quaternary Ammonium Compounds		*qac*	53(43.8%)			
MDR	63(52%)							
XDR	14(11.6%)							

MDR: multidrug-resistant, XDR: extensively drug-resistant

*n: Number of AmpC positive in *K. pneumoniae*

Out of 121, 52 (43%) showed resistance to ciprofloxacin and levofloxacin. Among the PMQR determinants, *qnrB* and *qnrS* were positive in 25 (48%) and 19 (36.5%) of the isolates, respectively. Of the *K. pneumoniae* isolates, 63 (52%) exhibited MDR. Of the isolates harboring PMQR, 51.9% had the same mutations in *gyrA* at amino acid 83 (replacement of serine with leucine) and 0% in *parC* at amino acid 80 (replacement of serine with isoleucine; GenBank accession no. HM068910). Class 1 integrons (97, 80.1%) were the most frequent integron class (Table 2).

### Carbapenem-resistant *K. pneumoniae* (CRKP) isolates

Overall, 51 (42.1%) of the CRKP isolates were resistant to carbapenems, 36 (70.5%) of which were MBL-positive. The average age of the CRKP patients was 64 and a half years. The most infectious CRKP samples were seen in respiratory (33, 64.7%), wound (8, 15.7%), urine (6, 11.7%), and blood infections (4, 7.8%). The isolates were all resistant to ampicillin, cotrimoxazole, cefixime, cefotaxime, ceftriaxone, ceftazidime, imipenem, and ertapenem (Table 3).

**Table 3:** Phenotypic and Genotypic antibiotic resistance rates in CRKP isolates

Antibiotic	CRKP isolates n:51	Resistance			Target gene	Frequency of resistance genes	Resistance	Target gene	Frequency of resistance genes
Ampicillin	51(100%)								
Cotrimoxazole	51(100%)	Sulfonamide			*sul1*	51(100%)			
					*sul2*	29(56.8%)			
Cefixime	51(100%)	ESBL+			*bla*_TEM_	51(100%)	n:51 AmpC+	*bla*_CIT_	28(54.9%)
Cefotaxime	51(100%)				*bla*_CTX−M1_	51(100%)		*bla*_CMY−2_	26(51%)
					*bla*_SHV_	51(100%)		*bla*_ACC_	4(7.8%)
Ceftriaxone	51(100%)				*bla*_CTX−M15_	49(96%)		*bla*_FOX_	0%
Ceftazidime	51(100%)				*bla*_CTX−M2_	0%		*bla*_MOX_	0%
					*bla*_CTX−M8_	0%		*bla*_DHA_	0%
Gentamicin	47(92.1%)								
Amikacin	26(50.9%)								
Tetracycline	27(52.9%)	Tetracycline			*tetA*	19(70.4%)			
Doxycycline	27(52.9%)								
Minocycline	22(43.1%)				*tetB*	11(40.7%)			
Tigecycline	2(3.9%)								
Ciprofloxacin	49(96%)	*Fluoroquinolone*			*gyrA*	28(57.1%)			
					*qnrB*	26(53%)			
Levofloxacin	49(96%)				*qnrS*	21(42.8%)			
					*qnrA*	13(26.5%)			
					*parC*	0%			
Ampicillin/sulbactam	51(100%)								
Piperacillin/tazobactam	51(100%)								
Imipenem	50(98%)	Carbapenemase		Ambler class A blactamases	*bla*_GES_	11(21.5%)			
					*bla*_KPC_	0%			
				Ambler class B metallo- β -lactamases (MBLs)	*bla*_NDM_	36(70.5%)			
					*bla*_VIM_	9(17.6%)			
Meropenem	51(100%)				*bla*_SIM_	0%			
					*bla*_GIM_	0%			
Doripenem	51(100%)				*bla*_IMP_	0%			
				Ambler class D b-lactamases	*bla*_Oxa−48_	46(90.1%)			
Ertapenem	51(100%)								
Aztreonam	50(98%)								
Colistin	4(7.8%)								
Fosfomycin	5(9.8%)								
		Integrase			*int1*	51(100%)			
					*int2*	39(76.5%)			
					*int3*	0%			
		Quaternary Ammonium Compounds			*qac*	48(94.1%)			
MDR	51(100%)								
XDR	14(27.4%)								

MDR: multidrug-resistant, XDR: extensively drug-resistant, CRKP: carbapenem-resistant *Klebsiella pneumoniae*

*n: Number of AmpC positive in CRKP isolates

All these isolates displayed MDR. The most common ESBL resistance genes were *bla*_TEM_ (51, 100%), *bla*_CTX−M−1_ (51, 100%), *bla*_SHV_ (51, 100%), and *bla*_CTX−M−15_ (49, 96%). The most abundant AmpC resistance genes were *bla*_CIT_ (28, 54.9%) and *bla*_CMY−2_ (26, 51%). The most common carbapenemase resistance genes were *bla*_Oxa−48_ and *bla*_NDM_ in 46/51 (90.1%) and 36/51 (70.5%) of the CRKP isolates, respectively (Table 3). A total of 31 isolates (60.7%) of CRKP were found with both *bla*_Oxa−48_ and *bla*_NDM_ genes together. Of the CRKP isolates, 3.9% were resistant to tigecycline. Amikacin was observed to be the most active factor among the aminoglycoside family of antibiotics. Tigecycline was the most effective antibiotic, with 96.1% susceptibility. *Bla*_NDM_-positive isolates were resistant to the most antibiotics, and all of them contained *bla*_TEM_, *bla*_CTX−M1_, *bla*_SHV_, and *bla*_CTX−M15_ resistance genes (Table 3).

## Discussion

These isolates were highly resistant to ampicillin (93.8%) and cotrimoxazol (64.4%) but least resistant to tigecycline (1.6%), colistin (3.3%), and fosfomycin (4.1%). In other studies in southern and northern Iran, *K. pneumoniae* isolates were most resistant to cefazolin (45.9%) and ampicillin/sulbactam (93%), respectively [23, 24]. In prior research in Tehran and southern Iran, these isolates showed the lowest antibiotic resistance to tigecycline (9% and 3.5%, respectively) [5, 23].

In this study, 64.4% of the *K. pneumoniae* isolates were resistant to cotrimoxazole, and the most abundant sulfonamide gene was *sul1* (70.5%). In other studies in Hamedan and Shahrekord, resistances to cotrimoxazole of 52% and 63%, respectively, were seen in *K. pneumoniae* isolates; the most abundant sulfonamide genes were *sul1* (60.9%) and *sul2* (60.6%), respectively [25, 26]. In this study, all CRKP isolates were resistant to cotrimoxazole, and all of these isolates exclusively contained *sul1* genes. In a study in Turkey, 86.3% resistance to cotrimoxazole was observed in CRKP isolates, and the most abundant sulfonamide gene was *sul1* (100%) [27].

It seems likeliest that the high use of cotrimoxazole in infections and the spread of resistance genes between bacterial strains have caused high resistance to cotrimoxazole and sulfonamides.

The genes most commonly associated with ESBL in *K. pneumoniae* isolates are *bla*_CTX−M_, *bla*_TEM_, and *bla*_SHV_ [28]. In this research, 63.6% of the *K. pneumoniae* isolates were ESBL, and the most common ESBL genes were *bla*_TEM_ (100%), *bla*_CTX−M1_ and *bla*_SHV_ (98.7%), and *bla*_CTX−M15_ (94.8%). In a study in Azerbaijan, 68% of the *K. pneumoniae* isolates were ESBL, and the most common ESBL genes were *bla*_SHV_ (58%) and *bla*_CTX−M15_ (55%). The widespread use of multiple β-lactam agents in recent years has led to the advent of ESBLs, which are frequent carriers of the genes *bla*_TEM_ and *bla*_SHV_ [24].

In this research, all CRKP isolates were 100% ESBL positive, and all of these isolates contained the genes *bla*_TEM_, *bla*_CTX−M1_, *bla*_SHV_ (100%), and *bla*_CTX−M15_ (96%). In other studies in Isfahan and northern Iran, 97.9% and 64% of CRKP isolates were ESBL positive, respectively, and the most abundant ESBL genes in these isolates were *bla*_CTX−M15_ (97.9%) and *bla*_SHV_ (91.4%), respectively [6, 24].

In this research, 96% of the CRKP isolates shared four of the same genes: *bla*_TEM_, *bla*_CTX−M1_, *bla*_SHV_, and *bla*_CTX−M15_. In another study, 93.6% of CRKP isolates shared three genes: *bla*_TEM_, *bla*_SHV_, and *bla*_CTX−M15_ [6]. These resistances are usually located on mobile genetic elements, such as plasmids, which are easily transferable within and between bacterial species. In the Middle East, studies have shown that the frequency of ESBL in *K. pneumoniae* has increased over the last 10 years [28].

In this report, 47.9% of the *K. pneumoniae* isolates were AmpC positive, and the most abundant genes were *bla*_CIT_ (48.3%) and *bla*_CMY−2_ (44.8%). In other studies, 9% and 19% of isolates were AmpC positive, and the most abundant AmpC genes were *bla*_MOX_ and *bla*_CMY_, respectively [29, 30].

In this report, all CRKP isolates were (100%) AmpC positive, and the most abundant genes were *bla*_CIT_ (54.9%) and *bla*_CMY−2_ (51%). In other studies, 100% and 45.9% of CRKP isolates were AmpC positive, and the most abundant gene was *bla*_DHA_ (100%) and *bla*_DHA_ (45.9%), respectively [31, 32].

MDR bacteria such as MDR *K. pneumoniae* is a common nosocomial pathogen that has become a global public health concern due to difficult-to-treat infections [2, 3, 33, 34]. In this investigation, 52% and 11.6% of the *K. pneumoniae* isolates were MDR and XDR positive, respectively. In other studies, 58% and 13% of *K. pneumoniae* isolates were MDR and XDR positive, respectively [24].

CRKP infection is c onsidered a significant threat to human health worldwide owing to rapid CRKP spread, a dearth of available therapeutic options, and the major impact of these infections on patient outcomes, including lengths of hospitalization, healthcare costs, and increased mortality [1, 35]. CRKP has caused many infection control problems in healthcare systems. The results of the CRKP outcomes in this study were troubling (42.1%). In studies in Isfahan and southwestern Iran, 33.7% and 55% of *K. pneumoniae* isolates were CRKP positive, respectively [36, 37].

There are differences in the frequency of CRKP and antibiotic resistance across Iranian studies. These differences may result from infection control policies carried out in the hospitals studied or the indiscriminate utilization of antibiotics.

Tigecycline and colistin are the last lines of antibiotic therapy against CRKP, and resistance to these drugs has become a major clinical challenge [3]. In this investigation, tigecycline, colistin, and fosfomycin were the most effective antimicrobial agents against CRKP isolates. Tigecycline resistance was 1.6%, while in other studies, this resistance rate was 9% or 0% [5, 6].

Globally, colistin-resistant CRKP has been recorded due to the increased use of colistin [3]. In this study, 0.6% colistin resistance was observed. In other studies, resistance to colistin has been reported to be 50% and 0% [5, 6]. Although colistin is efficient in remedying infections brought on by CRKP, colistin resistance is recognized to be induced during colistin remedying and can be brought on by genetic variations and mutations in chromosomal genes [38].

In this investigation, 1.6% of CRKP isolates had triple combination resistance to tigecycline, colistin, and fosfomycin. In other studies, 9% of CRE isolates had a combination resistance to both colistin and tigecycline [5].

This is the first study in central Iran in which *bla*_Oxa−48_ (46, 90.1%) and *bla*_NDM_ (36, 70.5%) were the most frequently indicated carbapenemases, which is consistent with findings elsewhere [5, 6]. The proximity between Turkey (where the first *bla*_Oxa−48_ gene was isolated), India (where the first *bla*_NDM_ gene was isolated), and Iran, frequent travel between the countries, and ease of resistance transfer among microorganisms are probably the reasons for the high isolation of MDR and CRKP strains in *bla*_Oxa−48_ and *bla*_NDM_ genes from Central Iran [24].

In this research, all CRKP isolates were MDR positive, and molecular analysis determined double or triple carbapenemase gene combinations (*bla*_Oxa−48_, *bla*_NDM_, and *bla*_GES_) with a co-existence of (*qnrB*, *qnrS*, and *qnrA*) genes.

In this research, *bla*_NDM_-positive isolates were resistant to the most antibiotics, and all of them simultaneously contained *bla*_TEM_, *bla*_CTX−M1_, *bla*_SHV_, and *bla*_CTX−M15_ resistance genes. In other studies, the simultaneous presence of these genes has also been observed [23, 39]. In our study, all of the *bla*_Oxa−48_- and *bla*_NDM_-producing CRKP isolates coharbored at least one ESBL gene, which is consistent with other reports [6].

In this research, 96% of the CRKP isolates were resistant to fluoroquinolone, and the highest fluoroquinolone resistance levels were obtained by *gyrA* (57.1%) and *qnrB* (53%). In other studies, 72.7% and 62.8% of the CRKP isolates were resistant to fluoroquinolone, and the highest fluoroquinolone resistance levels were obtained by *qnrS* and *gyrA* at 86.3% and 100%, respectively [27, 40].

Resistance rates to tetracycline and fluoroquinolone in CRKP isolates differ according to the indiscriminate use of these antibiotics, infection control policies, and their geographical distribution [40].

In this research, all CRKP isolates carried the *int1* gene. In other studies, *int1* was found in 91.4% of isolates in Sari and 81.6% in Hamadan [24, 25]. The most common integrons in clinical settings are classes I and II [24]. These differences could be due to levels of hygiene, microbial genetic diversity, and differences in the sources of samples [24].

## Conclusion

Undoubtedly, the emergence and spread of ESBL, AmpC, and carbapenemase genes in *K. pneumoniae* will further limit clinical therapeutic choices in Iran. Rapid and accurate diagnosis, appropriate isolation of patients with CRKP isolates, and strict, effective measures are crucial in controlling the infection and preventing the spread of these resistant isolates.

## Data Availability

The datasets analyzed and/or used during the current study are available from the corresponding author on reasonable request.
